# Prevalence of chronic chikungunya and associated risks factors in the French West Indies (La Martinique): A prospective cohort study

**DOI:** 10.1371/journal.pntd.0007327

**Published:** 2020-03-12

**Authors:** Antoine Bertolotti, Marême Thioune, Sylvie Abel, Gilda Belrose, Isabelle Calmont, Raymond Césaire, Minerva Cervantes, Laurence Fagour, Émilie Javelle, Catherine Lebris, Fatiha Najioullah, Sandrine Pierre-François, Benoît Rozé, Marie Vigan, Cédric Laouénan, André Cabié, the Chronic Chikungunya working group of University Medical Center of Martinique

**Affiliations:** 1 INSERM, CIC1410, CHU de la Réunion, Saint-Pierre, France; 2 CHU de la Réunion, service de maladies infectieuses-médecine interne-dermatologie, Saint Pierre, France; 3 CHU de Martinique, service de maladies infectieuses et tropicales, Fort-de-France, France; 4 Université des Antilles, EA 4537, Fort-de-France, France; 5 CHU de Martinique, Centre de ressource biologique de la Martinique, Fort-de-France, France; 6 INSERM, CIC1424, CHU de Martinique, Fort-de-France, France; 7 CHU de Martinique, laboratoire de virologie, Fort-de-France, France; 8 INSERM, IAME, UMR 1137; Université Paris Diderot, Paris, France; 9 INSERM, CIC-EC 1425, Hôpital Bichat, Paris, France; 10 Hôpital d’instruction des Armées Laveran, service de pathologie infectieuse et tropicale, Marseille, France; Aix Marseille Université, Institut de Recherche pour le Développement (IRD); Assistance Publique-Hôpitaux de Marseille, Microbes Vecteurs Infections Tropicales et Méditerranéennes (VITROME); Institut Hospitalo-Universitaire Méditerranée Infection, Marseille, France; 11 Département d’Épidémiologie, Biostatistique et Recherche clinique, Assistance Publique-Hôpitaux de Paris, Hôpital Bichat, Paris, France; Yale School of Public Health, UNITED STATES

## Abstract

**Background:**

The chikungunya virus (CHIKV) is a re-emerging alphavirus that can cause chronic and potentially incapacitating rheumatic musculoskeletal disorders known as chronic chikungunya arthritis (CCA). We conducted a prospective cohort study of CHIKV-infected subjects during the 2013 chikungunya outbreak in Martinique. The aim of this study was to assess the prevalence of CCA at 12 months and to search for acute phase factors significantly associated with chronicity.

**Methodology/Principal findings:**

A total of 193 patients who tested positive for CHIKV RNA via qRT-PCR underwent clinical investigations in the acute phase (<21 days), and then 3, 6, and 12 months after inclusion. The Asian lineage was identified as the circulating genotype. A total of 167 participants were classified as either with or without CCA, and were analyzed using logistic regression models. The overall prevalence of CCA at 12 months was 52.1% (95%CI: 44.5–59.7). In univariate analysis, age (RD 9.62, 95% CI, 4.87;14.38, p<0.0001), female sex (RD 15.5, 95% CI, 1.03;30.0, p = 0.04), headache (RD 15.42, 95% CI, 0.65;30.18 p = 0.04), vertigo (RD 15.33, 95% CI, 1.47;29.19, p = 0.03), vomiting (RD 12.89, 95% CI, 1.54;24.24, p = 0.03), dyspnea (RD 13.53, 95% CI, 0.73;26.33, p = 0.04), intravenous rehydration (RD -16.12, 95% CI, -31.58; -0.66 p = 0.04) and urea (RD 0.66, 95% CI, 0.12;1.20, p = 0.02) were significantly associated with the development of CCA. For the subpopulation with data on joint involvement in the acute phase, the risk factors significantly associated with CCA were at least one 1 enthesitis (RD 16.7, 95%CI, 2.8; 30.7, p = 0.02) and at least one tenosynovitis (RD 16.8, 95% CI, 1.4–32.2, p = 0.04).

**Conclusions:**

This cohort study conducted in Martinique confirms that CCA is a common complication of acute chikungunya disease. Our analysis emphasized the importance of age and female sex for CCA occurrence, and highlighted the aggravating role of dehydration during the acute phase. Early and adequate hydration were found to reduce the risk chronic chikungunya disorders.

**Trial registration:**

clinicaltrials.gov (NCT01099852).

## Author summary

Chikungunya is a tropical, mosquito-borne virus that has been re-emerging in the last decade. It has caused major epidemics in recent years, including in Reunion Island and in Southeast Asia. Nearly 2.5 billion people around the world are at risk of contracting the virus. In the acute phase of the illness, patients experience a flu-like syndrome with fever, headache, myalgia, rash, and severe arthralgia. These symptoms can persist for several months in some patients, and can lead to significant functional disability. During the 2013 epidemic in Martinique, we followed nearly 200 patients who had contracted chikungunya. More than half of the patients had a chronic form of the disease—mainly women over 50 years of age. Our statistical analyses indicate that poor hydration in the acute phase is a risk factor for developing chronic rheumatism. This suggests that, in the context of a chikungunya epidemic, patients should drink plenty of fluids as soon as the first symptoms appear.

## Introduction

Chikungunya virus (CHIKV) is a re-emerging alphavirus transmitted to humans by *Aedes* mosquitoes that has caused massive epidemics in Africa [1–3]; in the Indian Ocean islands [4–6]; in Southeast Asia [7,8]; in the Americas [9,10]; and in Italy, where the first European outbreak occurred in 2007 [11,12]. CHIKV is known to target human epithelial and endothelial cells, fibroblasts and macrophages [13–15], as well as human muscle satellite cells [16]. The virus is also suspected of neurotropism [17,18], which can lead to neurological complications [19–22].

Chikungunya, a Makonde term meaning “that which bends up” [23], is an acute illness similar to dengue that is characterized by the abrupt onset of high-grade fever, followed by constitutional symptoms such as polyarthritis, musculoskeletal pain, headache, and skin involvement [24], and in rare cases by severe manifestations such as encephalopathy [21,24], acute hepatitis [25,26], myocarditis [27–29], and multi-organ failure. The acute phase (viremic period) lasts from 5 to 10 days on average, and is resolved within a few weeks. In May 2015, the World Health Organization [30] defined a person with chronic chikungunya as a “person with previous clinical diagnosis of chikungunya after 12 weeks of the onset of the symptoms presenting with at least 1 of the following articular manifestations: pain, rigidity, or edema, continuously or recurrently.” Chronic arthralgia (i.e., “suspected chronic chikungunya arthritis” or “CCA”) is a common complication post CHIKV-infection. Some studies [31–37] have reported that 40 to 60% of patients with acute chikungunya experience significantly impaired quality of life in the long term. Other studies have found that patients affected by CCA were significantly older [31,33,38,39], were more likely to be female [6,31,39,40], had severe initial rheumatic symptoms [39,41,42], and had high CHIK-specific IgG titers [38,41]. Martinique is a French overseas department with a population of nearly 400,000 and located in the French West Indies. The first autochthonous cases of chikungunya were described in the region in November 2013 (in December 2013 in Martinique) [43]. The Asian lineage was identified as the circulating genotype [44]. At the end of the outbreak (in approximately January 2015), the number of affected people was estimated at 145,000 (36% of the population) [45]. The aim of the present study was: first, to determine the prevalence of CCA at 12 months of follow-up, and second, to search for acute phase factors significantly associated with chronicity.

## Methods

### Ethics statement

Ethical clearance was obtained from the French National Agency for the Safety of Medicines and Health Products (ANSM) (n°IDRCB 2010-A00282-37) and from the French Committee for the Protection of Individuals. Written and signed informed consent was obtained from all participants.

### CARBO presentation

In June 2010, the Department of Infectious Diseases at the Hospital of Fort de France initiated a descriptive and prognostic study of dengue in the French West Indies and French Guiana known as the “DAG” Study. This study was based on a hospital cohort of children and adults with suspected dengue and had as its main objective to identify the predictive factors of severe dengue. Following the introduction of CHIKV in Martinique, the inclusion criteria of the DAG study were extended to chikungunya cases, and the protocol was adjusted accordingly (namely by including a broad description of rheumatic disorders specific to chikungunya disease). In May 2014, the “DAG” study became the “DAG-2” study, and included a cohort composed of 2 groups: the dengue group and the chikungunya group. This study is registered on clinicaltrials.gov (NCT01099852). The DAG-2 study is still ongoing (the duration of follow-up is 36 months), and the present analysis was conducted at 12 months of follow-up.

At the beginning of 2016, the DAG-2 cohort was extended to other arboviruses (including the Zika virus), and was renamed “the CARBO cohort.”

This study was conducted at Pierre Zobda-Quitman Hospital in Fort de France, capital of Martinique, in the departments most likely to receive patients infected with CHIKV (the emergency department and the department of infectious diseases). As a result, the cohort of patients was mainly ambulatory and reflected the health care-seeking population affected by the epidemic in Martinique.

### Study population

Patients were included in the cohort between December 19, 2013, and December 4, 2014. Inclusion criteria were: age ≥ 16 years of age, ability to sign informed consent, blood sample positive for CHIKV RNA via qRT-PCR, and onset of symptoms (headache, arthralgia, myalgia, fever, rash, fatigue) ≤7 days.

### Data collection and follow-up

The DAG questionnaire was adapted to the objectives of the study. Data were collected at the initial visit (in the acute phase), and if possible on day 3, between days 5 and 7, and between days 8 and 10 after the onset of symptoms. The following data were recorded: sociodemographic data, comorbidities and clinical characteristics at baseline (headache, arthralgia, myalgia, fever, rash, fatigue, etc.), impact on quality of life as measured by the EuroQol 5 Dimensions (EQ5D) scale (S1 Fig), and treatments used. At the beginning of the epidemic, the medical team of the infectious disease department was trained by a group of rheumatologists, one of whom was a member of the CHIKV patient care team. Patients with CCA were managed in collaboration with the rheumatology department. For patients enrolled from July 2014 onwards (i.e., after adjustment of the protocol for the DAG-2 cohort), an accurate description of affected joints (location, swelling, stiffness, arthritis, enthesitis, joint pain, etc.), and an assessment of peripheral neuropathy with the *douleur neuropathique 4* (DN4, neuropathic pain 4) scale (S2 Fig) were also performed.

At 3 months (M3), patients were examined by a team physician to identify CCA cases. At 6 months (M6) and 12 months (M12), patients were interviewed by phone using the DAG questionnaire (which is made up of closed questions) to monitor persistent symptoms (in particular joint pain), the impact on quality of life (as measured by the “EQ5D” scale), and treatments received. Patients were definitively classified as having CCA if they answered NO at any time of follow-up from M3 onwards to the following question: “Do you feel completely recovered from CHIKV-infection, in other words, are you now free of joint pain, rigidity, or edema related to this infection?” A clinical examination to describe and manage remaining disorders was proposed to these patients. Only patients who felt completely recovered from chikungunya at all visits from M3 onwards were categorized as NO CCA (without chronic chikungunya disease). The global clinical assessment of CCA subjects was conducted using the “Multi-Dimensional Health Assessment Questionnaire” (MDHAQ). Patients with chikungunya are known to experience a fluctuation of their symptoms (continuously or recurrently) over time. Patients with relapsing CCA were defined as having “disorders present at 1 and/or 2 time points without recovery” and patients with lingering CCA were defined as having “disorders present at all 3 time points: M3, M6, and M12.”

The 12-month period of follow-up allowed for the comprehensive description of chronic chikungunya disorders and for patients to characterize the time point with the heaviest burden (M3, M6, or M12).

### Laboratory tests

Only CHIKV-viremic patients were recruited in the study. Blood cell counts and biochemical tests (C-reactive protein (CRP), ionogram, urea, creatinine and hepatic transaminases) were performed upon a blood sample testing positive for CHIKV RNA via qRT-PCR (RealStar Chikungunya RT-PCR kit 1.0—Altona Diagnostics, Hamburg, Germany). A biological bank was created but never used, and the viral load of patients was not collected.

### Data analyses and statistical tests

Results were expressed as mean and standard deviation or frequency (percentage), as appropriate. Comparisons between the no CCA group and the CCA group were performed using chi-square test, Fisher’s exact test, or Wilcoxon test, as appropriate. Results were reported as risk difference with 95% confidence interval.

A predictive set of factors associated with CCA were selected using the least absolute shrinkage and selection operator (LASSO) method, after multiple imputations with chained equations to address missing data for variable selection. The penalty term required by the LASSO method was selected using 5-fold cross-validation. Factors with less than 10% of missing data were considered for the analysis. Factors selected by LASSO have non-null coefficient. A final non-regularized logistic regression was conducted on factors with non-null coefficient, in which the effect of each factor was reported as an adjusted odds-ratio with 95% confidence interval.

All tests were two-sided, and *P* values of less than 0.05 were considered significant. All statistical analyses were performed with SAS 9.4 (SAS Institute, Cary, NC, USA).

## Results

**Fig 1:**
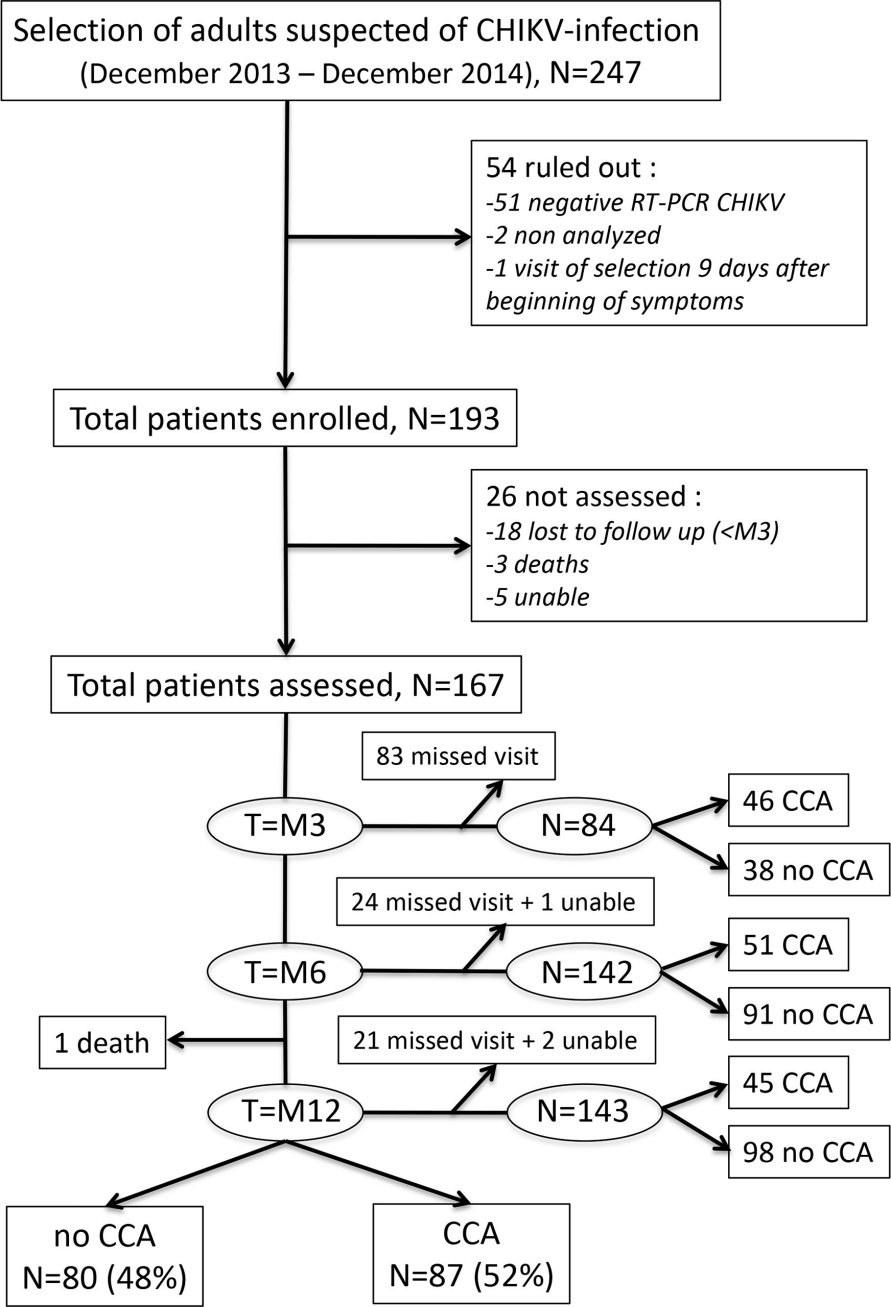
Diagram of the study population. “Unable”: patients unable to answer the question “Do you feel completely recovered from CHIKV-infection, in other words, are you now free of joint pain, rigidity, or edema related to this infection?”; “missed visit”: not present at a specific time point; “lost to follow-up”: patients who missed all follow-up visits from M3 onwards; “death”: dead patients; “CCA”: patients with chronic chikungunya arthritis; “no CCA”: patients without chronic chikungunya arthritis.

A total of 247 patients were eligible and consented to participate, 193 of whom were enrolled in the study (54 patients were excluded: 51 tested negative for CHIKV RNA via qRT-PCR, 2 were not tested for CHIKV RNA due to a temporary technical problem in the laboratory, and 1 was first evaluated more than 7 days after the onset of symptoms). The flowchart of the study is displayed in Fig 1.

### Overall prevalence of CCA at 12 months

Of the 193 patients, 21 were lost to follow-up (i.e., they did not attend any of the follow-up visits from M3 onwards), 3 of whom had died before M3 (causes of death were: adenocarcinoma of the prostate, multiple organ failure in the acute phase of CHIKV-infection, and unknown cause). Between M6 and M12, 1 patient died of myeloma; however, this patient was not excluded from the study because he had been reported as CCA at M3 (i.e., prior to his death). Five subjects had an undetermined CHIKC status at all follow-up visits, and were therefore excluded from the analysis.

A total of 167 patients were classified as either CCA or no CCA. Close to half the patients missed the M3 visit, as only 46 CCA patients and 38 no CCA patients were counted at the time. However, a greater number of patients were evaluated at M6 and M12, respectively 142 (85%; 51 CCA and 91 no CCA) and 143 (86%; 45 CCA and 98 no CCA) patients. Of the 167 participants, 87 had experienced CCA by M12, giving an overall prevalence of 52.10% (CI 95% 44.5–59.7). Ten CCA patients attended only 1 visit, and so their CCA profile (“relapsing” or “lingering”) could not be assessed. Of the 77 CCA patients, 64 (83.12%) had relapsing CCA and 13 (16.88%) had lingering CCA.

### Clinical data in the acute phase

The ratio of women to men was 1.80 (59 males and 106 females). Of the 167 included patients (age range from 20 to 91 years), 53.9% were aged over 50 years, with a mean (±SD) age of 51.35 (±16.10) years. Native American origins (n = 65, 38.92%) and Sub-Saharan African origins (n = 50, 29.94%) were the most represented. Cardiovascular and rheumatologic diseases were the most common pre-existing comorbidities: 36 (21.56%) participants had high blood pressure, 28 (16.77%) had healed fractures, 14 (8.38%) had osteoarthritis, 13 (7.78%) had diabetes, 13 (7.78%) had dyslipidemia, 11 (6.59%) had rheumatologic inflammatory diseases, and 9 (5.39%) had cancer.

The most frequently recorded clinical signs in the acute phase were fever (95.21%), intensity of pain > 4/10 on the visual analog scale (83.83%), headache (59.28%), myalgia (56.29%), and vertigo (31.74%); digestive symptoms (diarrhea, vomiting, and abdominal pain) were less common.

In the subpopulation with data on joint involvement in the acute phase (n = 73, including 37 no CCA and 36 CCA), the majority had articular disorders (69 patients, 94.52%) with joint pain (90.41%), swelling (42.46%), stiffness (27.40%), arthritis (20.55%), tenosynovitis (13.70%), and enthesitis (10.96%). The most affected joints were the ankles (68.49%), the wrists (58.90%), the distal interphalangeal joints of the hands (36.97%), the knees (50.68%), and the proximal interphalangeal joints of the hands (45.20%).

The clinical and biological characteristics of patients at the onset of disease are displayed in Table 1.

**Table 1:** Clinical characteristics and results of biological tests in CCA and no CCA adults at disease onset. DAG-2 study 2014–2016. Martinique (N = 167).

Variables	N	no CCA (%) (N_1_ = 80)	CCA (%) (N_2_ = 87)	Risk difference [95%CI]	p-value
DEMOGRAPHIC DATA					
**Mean Age**	**164**	**46.37 (15.83)**^#^	**55.99 (15.01)**^#^	**9.62 [4.87;14.38]**	**<0.0001**°
**Age**					<0.0001*
<35 years		22 (27.85)	4 (4.71)		
35–50 years		26 (32.91)	22 (25.88)		
51–65 years		22 (27.85)	42 (49.41)		
>65 years		9 (11.39)	17 (20.00)		
**Female**	**165**	**45 (56.25)**	**61 (71.76)**	**15.5 [1.03;30.0]**	0.04*
BMI (kg/m^2^)	161	26.63 (6.52)^#^	26.61 (5.91)^#^	-0.013 [-1.95;1.92]	1*
Employment	160	59 (75.64)	60 (73.17)	-2.47 [-15.99;11.05]	0.7*
CLINICAL SIGNS AT DISEASE ONSET					
Arthralgia	167	77 (96.25)	84 (96.55)	0.3 [-5.36;5.96]	1*
Fever	167	77 (96.25)	82 (94.25)	-2.0 [-8.42; 4.43]	0.7*
Visual analog scale (VAS) > 4	167	67 (83.75)	73 (83.91)	0.16 [-11.02;11.34]	1*
**Headache**	**167**	**41 (51.25)**	**58 (66.67)**	**15.42 [0.65;30.18]**	0.04*
Myalgia	167	46 (57.50)	48 (55.17)	-2.33[-17.38;12.72]	0.8*
Abdominal pain	167	28 (35.00)	27 (31.03)	-3.97 [-18.24;10.31]	0.6*
Rash	167	19 (23.75)	16 (18.39)	-5.36 [-17.74;7.02]	0.4*
Number of signs	167	3.27 (1.12)	3.40 (1.13)	0.13 [-0.22;0.47]	0.5°
OTHERS SIGNS IN THE ACUTE PHASE					
Dorsalgia	167	56 (70.0)	60 (68.97)	-1.03 [-15.01;12.94]	0.9*
Adenopathy	167	33 (41.25)	43 (49.43)	8.18 [-6.88;23.23]	0.3*
Asthenia	73	33 (89.19)	32 (88.89)	-0.3 [-14.64;14.03]	1*
Confinement to bed	94	24 (55.81)	36 (70.59)	14.77 [-4.63;34.18]	0.1*
Non-palmoplantar rash	94	26 (60.47)	29 (56.86)	-3.6 [-23.56;16.36]	0.7*
**Vertigo**	**167**	**19 (23.75)**	**34 (39.08)**	**15.33 [1.47;29.19]**	0.03*
Anorexia	73	23 (62.16)	24 (66.67)	4.50 [-17.43;26.44]	0.7*
Diarrhea	167	18 (22.50)	25 (28.74)	6.24 [-6.96;19.43]	0.4*
**Vomiting**	**167**	**9 (11.25)**	**21 (24.14)**	**12.89 [1.54;24.24]**	0.03*
Malaise	167	15 (18.75)	15 (17.24)	-1.51 [-13.18;10.16]	0.8*
Cough	167	16 (20.00)	12 (13.79)	-6.21 [-17.58;5.17]	0.3*
Nausea	167	7 (8.75)	16 (18.39)	9.64 [-0.59;19.87]	0.07*
**Dyspnea**	**167**	**14 (17.50)**	**27 (31.03)**	**13.53 [0.73;26.33]**	0.04*
Retro-orbital pain	73	6 (16.22)	10 (27.78)	11.56 [-7.28;30.41]	0.2**
Peripheral neuropathy	46	6 (23.08)	3 (15.00)	-8.08 [-30.60;14.44]	0.5**
Confusional syndrome	167	4 (5.00)	1 (1.15)	-3.85 [-9.13;1.42]	0.2**
Meningeal irritation	94	0	0		
Convulsions	94	0	0		
Coma	94	0	0		
Melena	95	0	1 (1.92)	1.92 [-1.81;5.66]	1**
Menorrhagia	167	1 (1.25)	0	-1.25 [-3.68;1.18]	0.5**
Petechial purpura	167	0	1 (1.15)	1.15 [-1.09;3.39]	1**
Pleural effusion	167	0	1 (1.15)	1.15 [-1.09;3.39]	1**
Shock	167	0	1 (1.15)	1.15 [-1.09;3.39]	1**
Cerebral failure	167	0	1 (1.15)	1.15 [-1.09;3.39]	1**
Low blood pressure (SBP < 90mmhg and DBP < 60mmhg)	155	4 (5.33)	7 (8.75)	3.42 [-4.60;11.43]	0.4**
High Blood pressure (SBP> 140mmhg and DBP > 90mmhg)	155	5 (6.67)	3 (3.66)	-2.92 [-9.93;4.10]	0.4**
Recoloration time >3 sec	147	9 (12.86)	4 (5.19)	-7.66 [-16.94;1.61]	0.1**
Pulse (beats per minute)	157	80.99 (19.09)^#^	76.96 (19.13)^#^	-4.02 [-10.05;2.00]	0.2°
Respiration frequency (breathing cycles)	131	18.42 (6.67)	19.48 (9.59)	1.07 [-1.77;3.91]	0.5°
Scale scores					
EQ5D scale score in the acute phase (total score out of 100)	144	60.38 (23.10)	56.05 (20.67)	-4.32 [-11.53;2.89]	0.2°
DN4 scale score (total score out of 10)	46	2.31 (1.89)	1.85 (1.56)	-0.46 [-1.51;0.60]	0.4°
MEDICAL HISTORY					
High Blood Pressure (HBP)	167	15 (18.75)	21 (24.14)	5.39 [-7.02;17.80]	0.4*
Healed fractures	167	15 (18.75)	13 (14.94)	-3.81 [-15.18;7.56]	0.5*
Allergy	167	6 (7.50)	9 (10.34)	2.84 [-5.77;11.46]	0.5**
Osteoarthritis	167	5 (6.25)	9 (10.34)	4.09 [-4.22;12.41]	0.3**
HIV	167	6 (7.50)	8 (9.20)	1.70 [-6.68;10.07]	0.7**
Diabetes	167	5 (6.25)	8 (9.20)	2.95 [-5.12;11.01]	0.5**
Dyslipidemia	167	5 (6.25)	8 (9.20)	2.95 [-5.12;11.01]	0.5**
Rheumatologic and inflammatory diseases	167	5 (6.25)	6 (6.90)	0.65 [-6.87;8.16]	0.9**
Cancer	167	4 (5.00)	5 (5.75)	0.75 [-6.09;7.58]	0.8**
Other immunosuppression (immunosuppressive therapy, chemotherapy)	167	2 (2.50)	5 (5.75)	3.25 [-2.72;9.22]	0.3**
Thrombopathy or chronic thrombocytopenia	167	1 (1.25)	6 (6.90)	5.65 [-0.21;11.50]	0.07**
Asthma	167	2 (2.50)	5 (5.75)	3.25 [-2.72;9.22]	0.4**
Dengue	167	4 (5.00)	2 (2.30)	-2.70[-8.42;3.02]	0.4**
Hemoglobinopathy	167	1 (1.25)	5 (5.75)	4.50 [-0.97;9.96]	0.2**
Rheumatologic and degenerative diseases	167	1 (1.25)	2 (2.30)	1.05 [-2.93;5.03]	1**
Other rheumatologic diseases	167	2 (2.50)	1 (1.15)	-1.35 [-5.44;2.74]	0.6**
Spondyloarthropathy	167	0	2 (2.30)	2.30 [-0.85;5.45]	0.5**
Lupus	167	1 (1.25)	1 (1.15)	-0.10 [-3.41;3.21]	1**
APS	167	1 (1.25)	0	-1.25 [-3.68;1.18]	0.5**
HBV	167	1 (1.25)	0	-1.25 [-3.68;1.18]	0.5**
HCV	167	0	0		
Rheumatoid polyarthritis	167	0	0		
Arthropathic psoriasis	167	0	0		
Psoriasis	167	0	0		
TREATMENTS					
Analgesic level 1	167	74 (92.50)	76 (87.36)	-5.14 [-14.20;3.92]	0.3*
Paracetamol	165	67 (83.75)	71 (83.53)	-0.22 [-11.51;11.07]	1*
Analgesic level 2	167	24 (30.0)	31 (35.63)	5.63 [-8.58;19.85]	0.4*
Antihistaminic	167	20 (25.0)	29 (33.33)	8.33 [-5.38;22.05]	0.2*
Non-steroidal anti-inflammatory drug (NSAID)	167	17 (21.25)	28 (32.18)	10.93 [-2.36;24.23]	0.1*
Phytotherapy (herbal medicine)	71	14 (37.84)	19 (55.88)	18.04 [-4.82;40.91]	0.1*
**Intravenous rehydration**	**99**	**13 (27.66)**	**6 (11.54)**	**-16.12 [-31.58;-0.66]**	0.04**
Analgesic level 3	167	0	1 (1.15)	1.15 [-1.09;3.39]	1**
Cumulative dose of paracetamol since the beginning of symptoms (milligrams)	83	5.57 (4.48)^#^	8.21 (7.48)^#^	2.63 [-0.15;5.41]	0.06°
BIOLOGY					
PT (Prothrombin Time) (%)	139	94.67 (27.03)^#^	94.23 (24.78)^#^	-0.44 [-9.13;8.82]	0.9°
ACT patient (seconds)	139	35.45 (3.99)^#^	35.49 (4.49)^#^	0.036 [-1.39;1.46]	0.9°
ACT control (seconds)	139	32.76 (0.59)^#^	32.68 (0.65)^#^	-0.07 [-0.28;0.13]	0.5°
Fibrinogen (G/L)	27	4.08 (1.20)^#^	3.83 (1.30)^#^	-0.25 [-1.24;0.74]	0.6°
Leukocyte (G/L)	160	4.98 (2.03)^#^	4.95 (2.41)^#^	-0.03 [-0.73;0.67]	0.9°
Red blood cells (G/L)	160	4.59 (0.59)^#^	4.46 (0.56)^#^	-0.14 [-0.32;0.04]	0.1°
Hemoglobin (G/dl)	160	13.29 (1.84)^#^	12.88 (1.47)^#^	-0.41 [-0.92;0.11]	0.1°
MCV (μm^3^)	160	87.68 (5.94)^#^	85.88 (13.61)^#^	-1.80 [-5.15;1.56]	0.3°
Neutrophils (G/L)	157	3.38 (1.75)^#^	3.39 (2.14)^#^	0.009 [-0.61;0.63]	1°
Eosinophils (G/L)	157	0.07 (0.09)^#^	0.05 (0.08)^#^	-0.02 [-0.04;0.01]	0.1°
Basophils (G/L)	157	0.02 (0.02)^#^	0.02 (0.02)^#^	0.002 [-0.005;0.008]	0.7°
Lymphocyte (G/L)	156	0.94 (0.47)^#^	0.97 (0.52)^#^	0.03 [-0.13;0.19]	0.7°
Monocyte (G/L)	157	0.44 (0.21)^#^	0.46 (0.22)^#^	0.02 [-0.04;0.09]	0.5°
Platelets (G/L)	159	204.60 (65.81)^#^	205.90 (69.56)^#^	1.3 [-19.97;22.64]	0.9°
Sodium (mmol/L)	151	137.60 (2.96)^#^	137.50 (2.65)^#^	-0.12 [-1.02;0.78]	0.8°
Potassium (mmol/L)	151	3.90 (0.44)^#^	3.91 (0.36)^#^	0.008 [-0.12;0.14]	0.9°
Chlorine (mmol/L)	151	87.94 (28.72)^#^	85.94 (30.81)^#^	-2.00 [-11.63;7.62]	0.7°
**Urea (mmol/L)**	**148**	**3.96 (1.22)**^#^	**4.62 (1.95)**^#^	**0.66 [0.12;1.20]**	**0.02**°
Creatinine (μmol/L)	156	83.39 (18.86)^#^	79.72 (23.08)^#^	-3.67 [-10.38;3.04]	0.3°
Protein (g/L)	31	75.14 (5.19)^#^	72.06 (6.35)^#^	-3.08 [-7.41;1.24]	0.2°
Calcium (mmol/L)	61	2.29 (0.10)^#^	2.29 (0.10)^#^	-0.004 [-0.06;0.05]	0.9°
Albumin (g/L)	11	39.90 (2.95)^#^	39.20 (3.47)^#^	-0.70 [-5.07;3.67]	0.7°
Total bilirubin (μmol/L)	157	9.07 (9.22)^#^	8.30 (6.67)^#^	-0.77 [-3.29;1.75]	0.5°
Conjugated bilirubin (μmol/L)	155	3.09 (2.32)^#^	2.84 (1.49)^#^	-0.26 [-0.87;0.36]	0.4°
AST (UI/L)	158	36.17 (48.42)^#^	31.18 (16.59)^#^	-5.00 [-16.10;6.11]	0.4°
ALT (UI/L)	158	27.19 (18.96)^#^	28.71 (18.79)^#^	1.53 [-4.42;7.47]	0.6°
GGT (UI/L)	156	38.88 (28.58)^#^	38.71 (42.99)^#^	-0.17 [-11.88;11.55]	1°
CK (UI/L)	133	206.90 (228.10)^#^	197.80 (218.10)^#^	-9.11 [-85.66;67.43]	0.8°
Lipase (UI/L)	14	26.00 (11.93)^#^	21.43 (1.00)^#^	-4.57 [-17.43;8.29]	0.5°
ALP (UI/L)	156	69.68 (43.11)^#^	69.04 (24.80)^#^	-0.65 [-11.61;10.31]	0.9°
Troponin (ng/mL)	34	5.87 (3.75)^#^	8.83 (9.00)^#^	2.96 [-1.68;7.59]	0.2°
CRP (mg/L)	153	25.57 (70.12)^#^	22.35 (30.89)^#^	-3.21 [-20.27;13.84]	0.7°
Lactates (mmol/L)	3	251.0	121.0 (169.7)^#^	-130.0 [-2770.7;2510.7]	0.6°
Hematocrite (%)	160	40.25 (5.15)^#^	38.98 (3.93) ^#^	1.28 [-0.15;2.70]	0.08°

*Chi-square test

**Fisher’s exact test

°Wilcoxon test

^#^Standard deviation; bold: significant variable; ^1^no CCA: 1 Sub-Saharan Africa + Western Europe, 1 Sub-Saharan Africa + Western Europe + Native America, 1 Sub-Saharan Africa + India, 1 Sub-Saharan Africa + Western Europe + India / CCA: 5 Sub-Saharan Africa + Western Europe, 2 Sub-Saharan Africa + India, 2 Sub-Saharan Africa + Western Europe + India; HIV: human immunodeficiency virus; HBV: hepatitis B virus; HCV: hepatitis C virus; DN4: *douleur neuropathique* 4/neuropathic pain 4; EQ-5D: EuroQol-5 dimension; APS: antiphospholipid syndrome; BMI: body mass index; SBP: systolic blood pressure; DBP: diastolic blood pressure; ACT: activated cephalin time; MCV: mean corpuscular volume; ALT: alanine aminotransferase test; AST: aspartate aminotransferase test; GGT: gamma-glutamyltransferase; CK: creatine kinase; ALP: alkaline phosphatase; CRP: C reactive protein.

### Identification of risk factors for chronic outcome (CCA)

**Fig 2:**
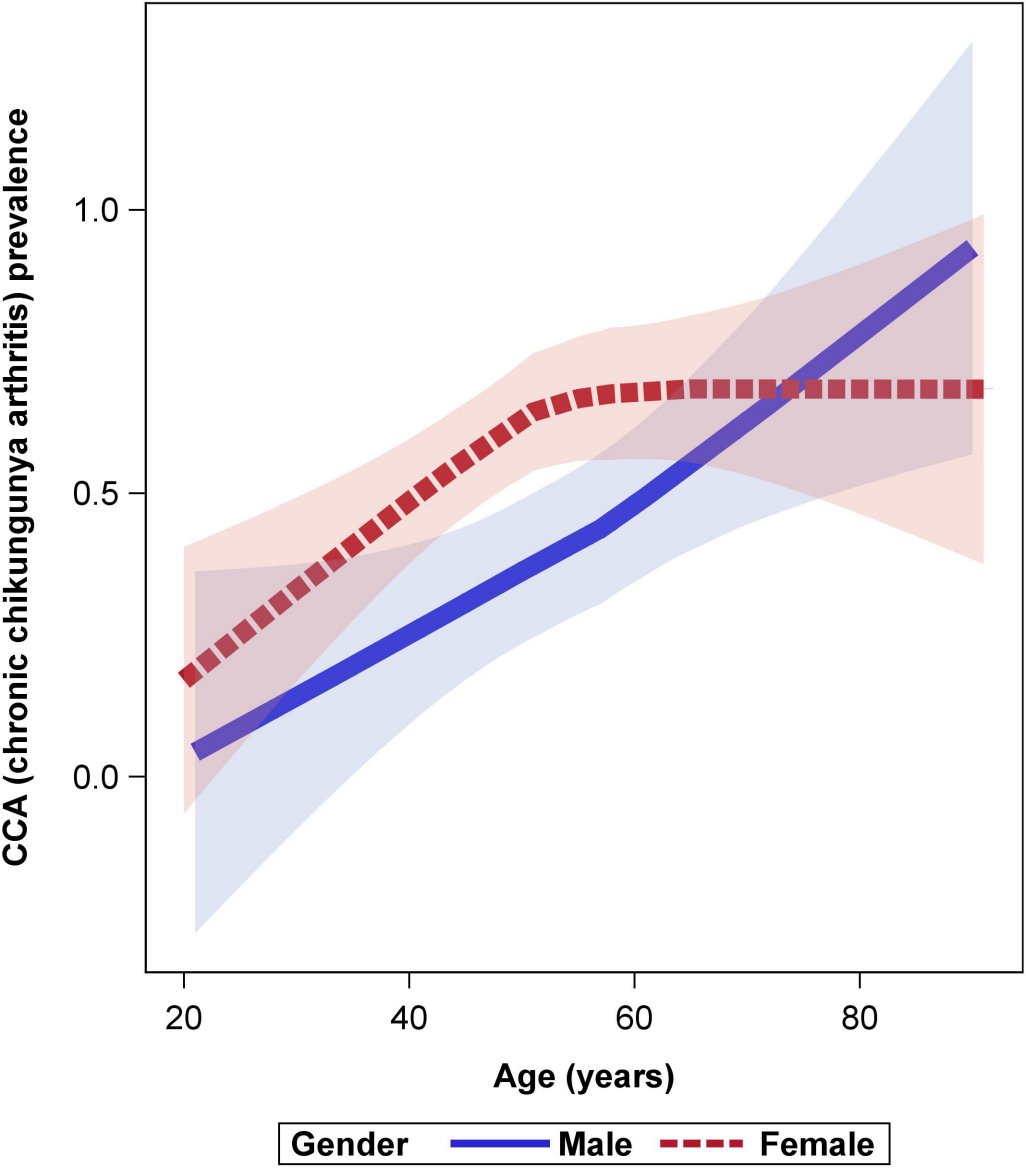
Loess curve between age and prevalence of chronic chikungunya according to sex. Pink corresponds to female sex and blue to male sex. DAG-2 study 2014–2016, Martinique.

Characteristics at disease onset in the 2 groups (no CCA and CCA) were compared to identify initial predictive factors for CCA. These characteristics are detailed in S1 and S2 Tables. Significant results are listed in Table 1. CCA patients were mainly women aged over 50 years (Fig 2). Overall, 58% (61/106) of the women developed CCA, versus 41% (24/59) of the men (sex data available for 165 patients). In univariate analysis (Table 1), the probability of being CCA increased with age (RD 9.62, 95% CI, 4.87;14.38, p<0.0001); female sex (RD 15.5, 95% CI, 1.03;30.0, p = 0.04); and some clinical signs at disease onset, namely headache (RD 15.42, 95% CI, 0.65;30.18 p = 0.04), vertigo (RD 15.33, 95% CI, 1.47;29.19, p = 0.03), vomiting (RD 12.89, 95% CI, 1.54;24.24, p = 0.03), dyspnea (RD 13.53, 95% CI, 0.73;26.33, p = 0.04), and intravenous rehydration (RD -16.12, 95% CI, -31.58; -0.66 p = 0.04). Biologically, the increase of urea (RD 0.66, 95% CI, 0.12; 1.20, p = 0.02) was significantly associated with the development of CCA. For the subpopulation with data on joint involvement in the acute phase, the risk factors significantly associated with CCA in univariate analysis were at least one enthesitis (RD 16.7, 95%CI, 2.8;30.7, p = 0.02) and at least one tenosynovitis (RD 16.8, 95% CI, 1.4–32.2, p = 0.04) (Table 2). Comorbidities (cardiovascular or rheumatologic diseases) were not found to be a risk factor for CCA (Table 1). Impact on quality of life, as measured by the EQ5D scale in the acute phase, was not significantly different between CCA and no CCA patients (Table 1).

**Table 2:** Joint involvement in the acute phase in a subpopulation of the DAG-2 study cohort 2014–2016, Martinique (N = 73): comparison between CCA and no CCA adults.

Variables	N	no CCA (%) (N = 37)	CCA (%) (N = 36)	Risk difference [IC95%]	P-value
**Sites**					
Ankles	73	26 (70.3)	24 (66.7)	-3.6 [-24.9;17.7]	0.7
Wrists	73	22 (59.5)	21 (58.3)	-1.1 [-23.7;21.5]	0.9
Distal interphalangeal joints (hands)	73	10 (27.0)	17 (47.22)	20.2 [-1.5;41.9]	0.07
Knees	73	18 (48.7)	19 (52.8)	4.1 [-18.8;27.1]	0.7
Shoulders	73	17 (46.0)	17 (47.2)	1.3 [-21.6;24.2]	0.9
Proximal interphalangeal joints (hands)	73	16 (43.2)	17 (47.2)	4.0 [-18.8;26.8]	0.7
Metacarpophalangeal joints	73	11 (29.7)	14 (38.9)	9.2 [-12.5;30.9]	0.4
Elbows	73	10 (27.0)	10 (27.8)	0.8 [-19.7;21.2]	0.9
Metatarsophalangeal joints	73	9 (24.3)	9 (25.0)	0.7 [-19.1;20.5]	0.9
Feet	73	9 (24.3)	8 (22.2)	-2.1 [-21.5;17.3]	0.8
Hips	73	7 (18.9)	8 (22.2)	3.3 [-15.2;21.8]	0.7
Hands	73	5 (13.5)	6 (16.7)	3.2 [-13.3;19.6]	0.7
Ribs	73	0	1 (2.8)	2.8 [-2.6;8.2]	0.5
Back	73	5 (13.5)	7 (19.4)	5.9 [-11.1;22.9]	0.5
Neck	73	10 (27.0)	13 (36.1)	9.1 [-12.2;30.3]	0.4
Rachis	73	16 (43.2)	10 (27.8)	-15.5 [-37.1;6.2]	0.2
Sternum	73	2 (5.4)	5 (13.9)	8.5 [-5.0;21.9]	0.2
Proximal interphalangeal joints (feet)	73	5 (13.5)	5 (13.9)	0.4 [-15.4;16.2]	1.0
Distal interphalangeal joints (feet)	73	4 (10.8)	5 (13.9)	3.1 [-12.0;18.2]	0.7
**Articular disorders**					
Joint pain	73	33 (89.2)	33 (91.7)	2.5 [-11.0;16.0]	0.7
Joint swelling without arthritis	73	17 (46.0)	14 (38.9)	-7.1 [-29.7;15.6]	0.5
Joint stiffness	73	7 (18.9)	13 (36.1)	17.2 [-2.9;37.3]	0.1
Arthritis	73	7 (18.9)	8 (22.2)	3.3 [-15.2;21.8]	0.7
Asymmetric joint involvement	73	7 (18.9)	5 (13.9)	-5.0 [-22.0;11.9]	0.6
**Tenosynovitis**	**73**	**2 (5.4)**	**8 (22.2)**	**16.8 [1.4;32.2]**	**0.04**
**Enthesitis**	**73**	**1 (2.7)**	**7 (19.4)**	**16.7 [2.8;30.7]**	**0.02**
Arthritis with synovitis	73	1 (2.7)	0	-2.7 [-7.9;2.5]	0.3
Periostitis	73	0	0		-

*test; bold: significant variable

Of the 77 chronic patients who answered at least 2 of the 3 questionnaires, 64 (83%) had relapsing joint symptoms and 13 (17%) had lingering joint symptoms. Moreover, 54 out of 87 CCA patients were clinically evaluated in the chronic phase (after adjustment of the protocol for the DAG-2 cohort), with joint pain (87%), stiffness (33.3%), and swelling (22%) being the most common chronic symptoms (Table 3). Enthesitis (13%), arthritis (7.4%), and tenosynovitis (5.6%) were less frequently observed. Ankles (31.5%) and knees (29.6%) were the most affected joints, while the proximal interphalangeal joints of the hands (24.1%), the wrists (22.2%), and the shoulders (22.2%) were less frequently involved. A quarter of patients presented peripheral neuropathy (27.7%), and 10 out of 30 patients had a mean MDHAQ score of 10.7. The mean EQ5D score was 51.56, indicating a significant decrease in quality of life relative to before CHIKV-infection (defined as 100/100).

**Table 3:** Clinical profile of CCA patients in the chronic phase between M3 and M12 (N = 54).

Variable	N	Value n (%)	min-max
Age: mean in years	48	49.86	21–91
Sex ratio Women/Men	53	2.78	
Number of joint assessments	54	7	0–32
Joint pain	54	47 (87.04)	
Joint swelling without arthritis	54	12 (22.00)	
Joint stiffness	54	18 (33.33)	
Arthritis	54	4 (7.41)	
Tenosynovitis	54	3 (5.56)	
Enthesitis	54	7 (12.96)	
Arthritis with synovitis	54	1 (1.85)	
Periostitis	54	1 (1.85)	
Peripheral neuropathy	47	13 (27.66)	
DN4 score mean value in the chronic phase	47	2.2	0–7
EQ5D score mean value in the chronic phase: mean points	49	51.56	1–100
MDHAQ score mean value in the chronic phase: mean points	43	10.7	0–22.7
Sites of assessment			
Ankles	54	17 (31.48)	
Knees	54	16 (29.63)	
Proximal interphalangeal joint (hand)	54	13 (24.07)	
Wrists	54	12 (22.22)	
Shoulder	54	12 (22.22)	
Metacarpophalangeal joint	54	10 (18.52)	
Metatarsophalangeal joint	54	9 (16.67)	
Elbow	54	7 (12.96)	
Distal interphalangeal joint (hand)	54	7 (12.96)	
Hand	54	3 (5.56)	
Foot	54	3 (5.56)	
Hip	54	3 (5.56)	
Distal interphalangeal joint (foot)	54	2 (3.70)	
Proximal interphalangeal joint (foot)	54	1 (1.85)	

DN4: *douleur neuropathique* 4/neuropathic pain 4; EQ-5D: EuroQol-5 dimension; MDHAQ: multidimensional health assessment questionnaire.

A multivariable analysis performed using the LASSO method is presented in S1 Table. Age (aOR 1.08 95%CI, 1.05–1.12, p<0.0001), female sex (aOR 2.44 95%CI, 1.03–5.88, p = 0.046), vomiting (aOR 3.37 95%CI, 1.06–11.8, p = 0.045), and Low blood pressure (aOR 6.67 95%CI, 1.47–36.6, p = 0.02) were identified as the mains risk factors for CCA.

## Discussion

This was the first prospective cohort study of CHIKV-infected cases to be conducted in Martinique during the 2013-outbreak. The overall prevalence of chronic chikungunya in our cohort was 52.10% (CI 95% 44.50–59.70), which confirms that it is a common sequela after acute infection. Indeed, previous studies have reported prevalences ranging between 53.70% and 87.20% globally [6,31–33]—except in India, where prevalences have been shown to vary between 4.10% and 7.00% [46,47] (possibly due to viral or population specificities).

During previous outbreaks, age over 50 and female sex were shown to be the main risk factors for chronic chikungunya disease [6,32,34,40,41,48,49]. Some studies focusing on gender have suggested that the menstrual cycle [50] and ovulation influence the production of pro‐inflammatory cytokines by monocytes [50,51] and that estradiol plays a role in the increase of antibody production [52,53]. However, these hypotheses have not been fully tested.

In our study, increased urea (p = 0.02) and reduced frequency of intravenous rehydration (p = 0.04) were associated with the development of CCA in univariate analysis. This finding suggests that initial dehydration is one of the potential factors leading to the development of CCA, just as it can cause complications in other arbovirus infections [54,55]. Our results also indicate that vertigo (p = 0.03) and headache (p = 0.04) are symptoms of dehydration, and that vomiting (p = 0.03) is an aggravating factor for dehydratation. Note that vomiting was a significant factor in the multivariable analysis, along with age and sex. The difficulty in diagnosing dehydration and the lack of human and material resources explain why rehydration was not systematically performed in our cohort of patients with CHIKV.

Because cartilage is largely composed of water [56], dehydration may result in its degradation by causing cracks and increasing fragility [57,58]. A study by Sahni et al. [59] on the impact of prolonged dehydration on osseous tissue and calcium phosphate metabolism in mice concluded that prolonged water deficiency leads to decreased bone mass, with an increased rate of bone resorption and mineralization defect. It is also established that calcium metabolism disorders stimulate parathyroid hormone (PTH) secretion and activation of bone resorption by osteoclast. In our study, no difference in calcemia was observed between CCA and no CCA patients in the acute phase. However, the rate of calcium was available for only 61 patients (as our study sample was small), and this one-off measure does not reflect metabolic disorders over the medium to long term. In fact, hydration may help dilute pro-inflammatory cells and cytokines recruited in joints and may enhance anti-inflammatory response. While articular cartilage is a mechanically highly challenged material with very limited regenerative ability, recent data by Boettcher et al. [60] have shown that a broad range of mechanical and structural properties of cartilage can be restored after rehydration with physiological salt solution.

CHIKV has been shown to damage cartilage [61] and synovial tissue [62]. In view of this, we make the hypothesis that patients suffering from dehydration at the onset of the disease are at higher risk of subsequently developing damaged cartilage, which may lead to more severe joint injury, slower recovery, and chronic injury. While it has been suggested that the intensity of symptoms at presentation is predictive of CCA [41], our results do not allow us to formally respond to the hypothesis we have formulated: dehydration during the acute phase of CHIKV infection is a cause of CCA. Indeed, we did not report on the occurrence of dehydration per se. Future studies should explicitly test this hypothesis (i) by accurately quantifying the volume of liquid ingested by patients during the acute phase in prospective studies; (ii) by conducting a randomized clinical trial comparing high intravenous and oral rehydration versus rehydration advice with subsequent quantification. Lastly, dehydration may contribute to the increase in viremia through serum concentration, as some studies have reported higher CHIKV plasma viral loads in CCA patients [62,63]. However, we did not investigate this parameter in our study due to a lack of funding.

Contrary to other authors, we found no association between CCA and pre-existing diseases—whether rheumatic disorders [48] like osteoarthritis [64] and rheumatoid arthritis [65–69] or cardiovascular diseases like hypertension [70] and diabetes [34].

Some studies have described the inflammatory and autoimmune processes in the acute and chronic phases of chikungunya disease [62,71]. Several of them have identified a positive relationship at disease onset between CRP level [34,65], pro-inflammatory cytokines [62,71], and severity at presentation [41,42,64]. However, no autoimmune markers for CHIKV have been highlighted in observational studies [34]. In other studies, biomarkers associated with CCA were IL-6 secretion [63] and GM-CSF at disease onset [62,63]. In a study conducted in Reunion Island, the proper role of specific IgG titers in predicting CCA was observed and positively correlated with age, female sex, and severity of initial rheumatic symptoms [41]. Yet, this study found no association between CRP level at disease onset and CCA.

The main clinical characteristics of chronic patients in our study were consistent with previous descriptions [8,24,38]: polyarthralgia, articular stiffness, swollen joints, and arthritis. Moreover, as was the case in previous studies [38,72–74], enthesitis and tenosynovitis were observed in our cohort. One study reported relapsing arthralgia for the majority of examined patients [40]. In our study, the development of CCA was associated with at least 1 tenosynovitis or 1 enthesitis in the acute phase. These 2 conditions, which are highly specific to symptomatic CHIKV infection, have not yet been identified as risk factors for CCA [75–77].

Some studies have highlighted the impact of CCA on daily activities and quality of life [35–38,41]. Patients suffering from CCA have been shown to require a multidisciplinary medical care program [78,79]. One study recommends long-term follow up by a general practitioner, often in cooperation with a rheumatologist or a rehabilitation physician [80]. Chikungunya disease and especially CCA with relapsing sequela have been shown to induce anxiety, stress, and even a nervous breakdown [81,82]. Medical treatments include analgesics, anti-inflammatory drugs, corticosteroids, and in severe cases anticonvulsant and immunosuppressive therapy [79,83,84]. Moreover, treatment may be combined with physiotherapy, physical therapy, and psychiatric and psychological evaluation [81,82–85]. Long-term disabilities caused by chikungunya are costly at the individual and collective scales. The economic impact of the 2005–2006 chikungunya outbreak in Reunion Island was estimated at 51.63 million US dollars [86].

One of the strengths of our study was the early inclusion and prospective follow-up of patients located at the epicenter of the epidemic. Another strength was that patients were examined physically by a clinician investigator at disease onset and at M3 to monitor clinical manifestations. This objective assessment of symptoms probably prevented an overestimation of symptoms on the part of patients, though some data (e.g., those collected via joint pain assessment) remain subjective.

Our study also has limitations that must be acknowledged. First, the phone interviews conducted at M6 and M12 may have led to a declarative bias. The rate of lost to follow-up was high, though phone calls helped to reduce it. Second, the study cohort did not fully reflect the population affected by the epidemic because subjects were not sampled from the general population; thus, data for elders may be missing as their symptoms are atypical and easily misdiagnosed [87,88]. Third, the plasma viral load of CCA and no CCA patients could not be measured due to a lack of funding; however, future studies could exploit our biological bank to assess this variable and to conduct other immunological and virological investigations.

To conclude, this first prospective study conducted during the chikungunya outbreak in Martinique has described the course of the disease in the local population and has estimated the burden at 1 year. Our findings confirm that more than half of adult symptomatic infections become chronic, with age and female sex as major risk factors for incident chronic chikungunya. The predictors of chronicity identified in the acute phase lead us to suggest that dehydration is a compounding factor in the occurrence of persisting chikungunya-associated rheumatic disorders. This hypothesis opens up new avenues for prevention and research in CHIKV pathways.

## Supporting information

S1 Fig EuroQol 5 Dimensions (EQ5D) score, *in French*. (DOCX)

S2 Fig Neuropathic pain score (DN4), *in French*. (DOCX)

S1 Table Significant characteristics of CCA and no CCA adults at disease onset. Multivariable analysis using least absolute shrinkage and selection operator. DAG-2 study 2014–2016, Martinique (N = 167).
(DOCX)

S2 Table STROBE Statement—Checklist of items that should be included in reports of cohort studies. (DOC)
