# Supplementary figures and images for: Prevalence of chronic chikungunya and associated risks factors in the French West Indies (La Martinique): A prospective cohort study
Source: PLoS Negl Trop Dis. 2020 Mar 12;14(3):e0007327. doi: 10.1371/journal.pntd.0007327 (PMC7100975; doi:10.1371/journal.pntd.0007327)

***
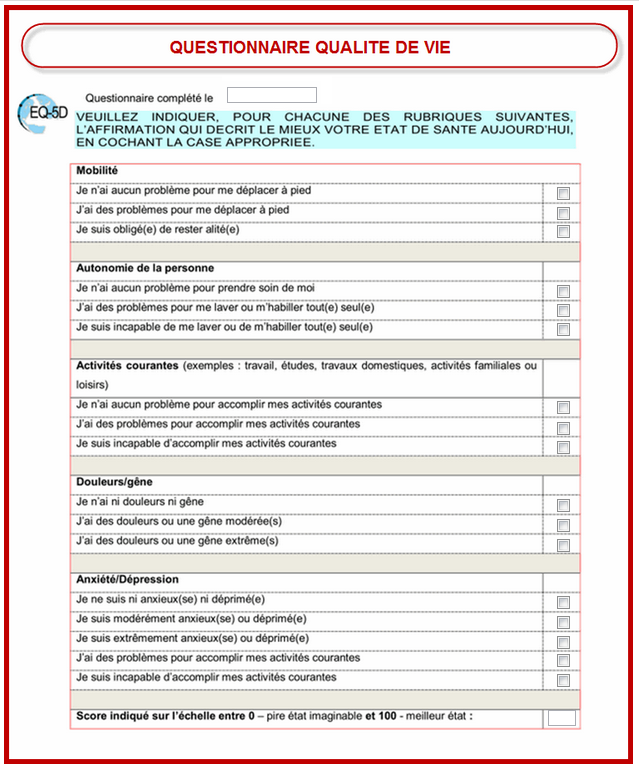
***


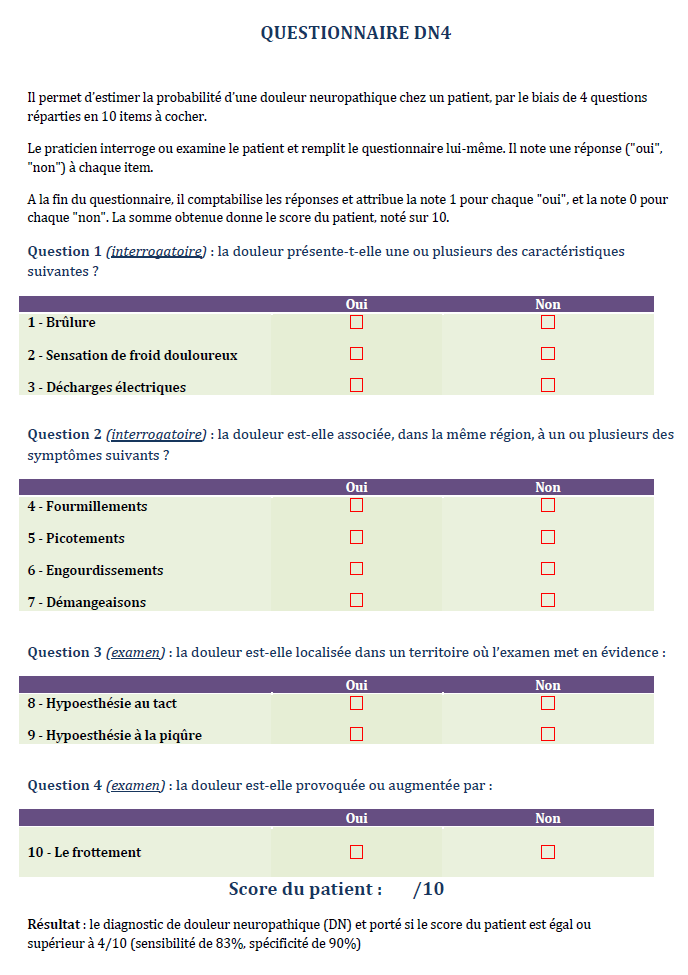

Supplement: S1 Fig — (DOCX) [file pntd.0007327.s001.docx]

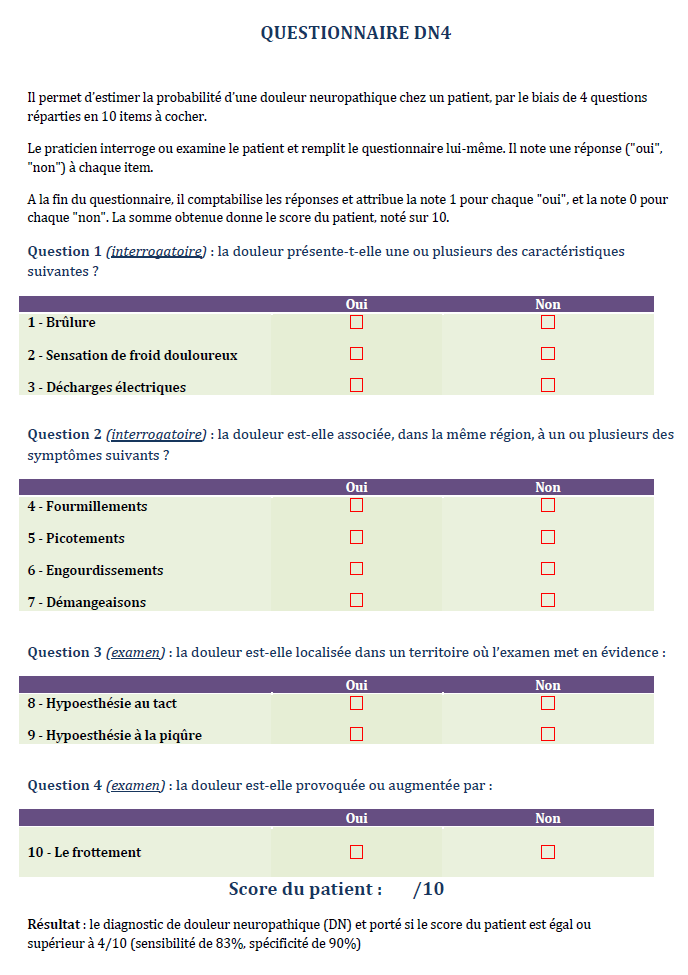

Supplement: S2 Fig — (DOCX) [file pntd.0007327.s002.docx]
