# Supplementary material for: Prevalence of chronic chikungunya and associated risks factors in the French West Indies (La Martinique): A prospective cohort study
Source: PLoS Negl Trop Dis. 2020 Mar 12;14(3):e0007327. doi: 10.1371/journal.pntd.0007327 (PMC7100975; doi:10.1371/journal.pntd.0007327)
Supplement: S1 Table — Multivariable analysis using least absolute shrinkage and selection operator. DAG-2 study 2014–2016, Martinique (N = 167). (DOCX) [file pntd.0007327.s003.docx]

**S3 Table. Characteristics of CCA and no CCA adult at disease onset. Multivariable analysis using least absolute shrinkage and selection operator. DAG-2 study 2014-2016, La Martinique (N=167).**

| **Variables** | **aOR [95%CI]** | **p-value** |
| --- | --- | --- |
| DEMOGRAPHIC DATA |  |  |
| Mean Age (sd) | 1.08 [1.05 – 1.12] | <0.0001 |
| Female | 2.44 [1.03 – 5.88] | 0.046 |
| CLINICAL SIGNS AT DISEASE ONSET |  |  |
| Fever | 0.27 [0.004 – 1.69] | 0.17 |
| Headache | 1.88 [0.79 - 4.55] | 0.16 |
| Myalgia | 0.56 [0.22 – 1.33] | 0.19 |
| OTHERS SIGNS AT THE ACUTE STAGE |  |  |
| Adenopathy | 2.06 [0.88 – 5.02] | 0.10 |
| Vertigo | 2.37 [0.96 – 6.06] | 0.06 |
| Vomiting | 3.37 [1.06 – 11.8] | 0.045 |
| Nausea | 2.45 [0.71 – 9.30] | 0.17 |
| Dyspnea | 1.64 [0.57 – 4.88] | 0.36 |
| Confusional syndrome | 0.59 [0.002 – 6.74] | 0.69 |
| Menorrhagia | - | 0.99 |
| Low blood pressure  (SBP < 90 mmhg and DBP < 60mmhg) | 6.67 [1.47 – 36.6] | 0.02 |
| Recoloration time >3 sec | 0.39 [0.008 – 1.74] | 0.23 |
| Pulse (beats per minute) | 0.99 [0.97 – 1.01] | 0.22 |
| MEDICAL HISTORIES |  |  |
| Thrombopathy or chronic thrombopenia | 1.85 [0.21 – 41.7] | 0.62 |
| Asthma | 8.74 [0.98 – 112.9] | 0.07 |
| Hemoglobinopathy | 2.09 [0.25 – 44.7] | 0.54 |
| Spondylarthropathy | - | 0.99 |
| SAPL | - | 0.99 |
| TREATMENTS |  |  |
| Analgesic level 1 | 0.42 [0.01 – 1.68] | 0.22 |
| BIOLOGICAL |  |  |
| VGM (µm3) | 0.95 [0.89 - 1] | 0.10 |
| Platelets (G/L) | 1.0 [0.99 – 1.01] | 0.23 |
| TGP (UI/L) | 1.0 [0.98 – 1.03] | 0.49 |
